# Identifying people with tuberculosis and linking to care: finding the missing millions — meet the guest editors

**DOI:** 10.1186/s44263-023-00006-5

**Published:** 2023-07-31

**Authors:** Rachael M. Burke, C. Finn McQuaid

**Affiliations:** 1 Malawi Liverpool Wellcome Trust Clinical Research Programme, Blantyre, Malawi; 2 Clinical Research Department, Faculty of Infectious and Tropical Disease, London School of Hygiene & Tropical Medicine, London, UK; 3 TB Modelling Group, TB Centre and Centre for Mathematical Modelling of Infectious Diseases, Department of Infectious Disease Epidemiology, London School of Hygiene & Tropical Medicine, London, UK

## Abstract

In this Q&A, Rachael Burke and Finn McQuaid answer questions about their research fields and share their experiences of guest-editing the journal’s collection on identifying people with tuberculosis and linking to care.

## What is the focus of your research and what drew you to this field?

*Rachael M. Burke*: I spent a quite a lot of years at the early stage of my career trying to figure out whether I should be an “HIV researcher” or a “TB researcher”, and it was only later as I settled into my role that I realised I didn’t have to choose and I could do both.

I’m an infectious disease physician (specialist registrar in UK terms) with research training in clinical epidemiology and clinical trials. My focus is around diagnostics and randomised evaluations — our research group has done several trials of non-pharmaceutical interventions such as new TB diagnostics, HIV self testing, active case finding for TB, anti-retroviral therapy (ART) and TB timing.

HIV-associated TB is my major interest, but I have done work around improving care for seriously unwell people living with HIV (many of whom have TB [1], and TB is too often undiagnosed in this group) and TB active case finding in the general population (many of whom also need equitable access to HIV testing and care [2]).

I was initially drawn to HIV and TB research through a conviction that there was substantial injustice in access to and quality of healthcare, and was particularly moved by campaigning around access to ART medicines in the early 2000s when I was a teenager. Since then I have developed more skills and experience — I have completed medical school, spent years in postgraduate medical education, done a Masters in Global Health and submitted a PhD in clinical epidemiology. However, I am still trying to “join the dots” together and work out how to use my skill set as a physician and epidemiologist to be an ally and advocate for health justice, particularly as it relates to HIV and TB.

*C. Finn McQuaid*: I am a mathematical modeller with a research focus on TB epidemiology. I am particularly interested in the prevention and care of drug-resistance [3]. Globally ~30% of antimicrobial resistance-related deaths are thought to be due to drug-resistant (DR) TB, with approximately half a million people suffering from drug-resistant disease annually [4]. However, only 4 in 10 people with DR-TB are enrolled in treatment, and just over half of those are successfully treated [5]. Mortality and treatment failure is particularly high, where treatment requires the use of expensive, toxic drugs for 6 or more months, placing a significant burden on both the patient and health system provider [6].

At the same time, 3 in every 1000 people globally have DR-TB infection, and this figure is rising [7]. Left untreated, up to 10% of these (over 2.3 million people worldwide) could progress to DR-TB disease. To achieve DR-TB elimination, and address the three pillars of the End TB Strategy (reducing TB deaths, TB incidence and catastrophic costs faced by TB-affected families due to TB), DR-TB needs to be correctly identified and treated, and appropriate preventive therapy (treatment to reduce the risk of developing future disease) employed for DR-TB infection.

## What are the main problems that need addressing in the field?

*RMB*: To be honest, I think the underlying problems are impoverishment and apathy. Drugs, vaccines and screening programmes are wonderful and people should have access to them. I am from Ireland, and TB was eliminated as an Irish public health problem before even effective antibiotics were discovered.

But in terms of problems that might be (slightly) more tractable by researchers, I think the two major issues are diagnostics for TB and access to healthcare for TB and other diseases. The diagnostic tools for TB remain inadequate. We need a suite of diagnostic tools that are suitable for different scenarios — it is likely that the diagnostic tools needed to maximise benefits and minimise harms for TB screening are different from the diagnostic tools need to diagnose TB in children and different again from what you need to diagnose TB in seriously unwell adults living with HIV. Some progress is being made for example using Artificial Intelligence in Chest X-ray interpretation for TB screening, but there have been some setbacks too such as disappointing field performance of newer urine LAM tests.

Testing needs to be set in the context of access to healthcare. A few years ago, I led a systematic review of the population-level impact of TB screening (or active case finding) [8]. In some of the studies included in that review, it was, frankly, difficult to fathom how people in the “control” arms ever got a TB diagnosis — often descriptions were of remote areas with long distance to primary healthcare centres and the primary healthcare centres didn’t have access to TB testing. In that case, no wonder a mobile TB X-ray unit and molecular lab in a van increased TB diagnoses! Everyone — and particularly those who are poor or otherwise vulnerable — need equitable access to primary care and high-quality TB diagnostics. Whilst community-based TB screening likely has its place, it’s not a substitute for accessible primary healthcare.

*CFM*: One of the key issues in the field remains the closing of the gap between the large number of people who we know have DR-TB disease, and those who are able to access diagnosis (including drug-susceptibility testing) and care. This has been further exacerbated by COVID-19, with more people than ever suffering from undiagnosed DR-TB [3]. Further, there is a real need to improve treatment success rates, including through identifying those at an increased risk of poor treatment outcomes and providing them with additional support.

## How do you expect this field to develop in the next few years?

*RMB*: I would love to see more work about people and communities’ values and preferences regarding TB diagnostics, and about individual-level benefits and harms of TB screening programmes. I am really excited to see emerging research about host-pathogen biology of sub-clinical/minimal TB disease states, and about the impact of subclinical TB on transmission. But I think before we run headlong into screening large numbers of people for subclinical TB, it is imperative to have much more information on the individual (as well as community) level balance of benefits and harms of screening.

The other part of my work is focused right on the other “end” of the TB disease spectrum from community-based screening for subclinical TB — at improving diagnosis of TB in people living with HIV who are seriously unwell or in hospital. I hope that over the next few years, we will see more work in diagnostics, clinical decision algorithms and perhaps new treatment strategies for people with disseminated TB and who are at extremely high risk of death.

*CFM*: In the coming years we are likely to see an increased focus on identifying and supporting those who are at an increased risk of both disease and poor treatment outcomes, including important comorbidities and risk factors such as undernutrition (to which 20% of TB is attributable [3]). Critical research is needed to develop optimal targeted case finding and support interventions and delivery mechanisms, including those outside the traditional biomedical (such as nutritional support and cash transfer schemes). There are also likely to be large changes with the availability of new TB vaccines, which will have major repercussions across the field, including for how programmes choose to spend their limited budgets.

## What are you most excited about in your role as a guest editor for this collection?

*RMB*: As a relatively junior researcher I’ve been really excited to be guest editor for this collection. It has been wonderful to connect with people in the field whose work I’ve admired from a distance. I’ve enjoyed correspondence with authors and getting to work closely with my wonderful co-guest editor Finn.

I hope the special issue is thought-provoking and contains both great research articles, and commentaries putting this topic in context.

*CFM*: Thinking through the different aspects of what we at first thought was a relatively straightforward topic — it was great to brainstorm amazing people at the forefront of the field who we think will have interesting and novel things to say, and to invite them to contribute. I have also enjoyed seeing how many of them have said yes! I look forward to this collection receiving many manuscripts on the topic, which advance our understanding of both what works in finding the missing millions, and how a changing natural history paradigm and research landscape affect these approaches.

## Data Availability

Not applicable
